# A systematic study of mitigation strategies to prevent the emission of malodours from primary settlers in wastewater treatment plants

**DOI:** 10.1007/s00449-026-03335-5

**Published:** 2026-05-20

**Authors:** Nataliia Kurnikova, Manuel Toledo, Raúl Muñoz

**Affiliations:** 1 Institute of Sustainable Processes, University of Valladolid, Paseo del Prado de la Magdalena, 3-5, Valladolid, 47011 Spain; 2 Department of Chemical Engineering and Environmental Technology, School of Industrial Engineering, University of Valladolid, C/ Dr. Mergelina, s/n, Valladolid, 47011 Spain; 3 Departamento de Química Inorgánica e Ingeniería Química, Área de Ingeniería Química, Universidad de Córdoba, Instituto Químico para la Energía y el Medioambiente (IQUEMA). Campus de Excelencia Internacional Agroalimentario (ceiA3), Campus Universitario de Rabanales (N-IV, km 396), edificio Marie Curie (C3), 14071 Córdoba, Spain

**Keywords:** Hydrogen sulfide, Methyl mercaptan, Odor mitigation strategies, Primary settler, VSC, WWTP

## Abstract

Odor emissions from wastewater treatment plants (WWTPs), primarily driven by sulfur-containing compounds, represent a major environmental and operational challenge that requires effective mitigation strategies. The present study assessed the effectiveness of different strategies of sulfur-borne odor mitigation in the primary settler of a pilot-scale WWTP. The most effective technique was the dosing of the precipitating agent FeCl_3_, which showed removal efficiencies (REs) of 88.5 ± 7.3% for H_2_S and 63.3 ± 3.5% for methyl mercaptan (CH_3_SH), using an optimal Fe (III): S molar ratio of 2:1 based on the influent characteristics of the primary settler. The addition of oxidants (H_2_O_2_, NaClO) in the primary settler demonstrated a low efficiency in mitigating sulfur-containing gas emissions, potentially due to the occurrence of scavenging reactions. However, chemical oxidation with sodium hypochlorite (NaClO) exhibited a strong oxidative capacity, achieving a 98.2 ± 1.7% RE of total dissolved sulfides (S^2−^) at an estimated effective molar ratio of 16:1 (Cl: S). Biological oxidation of odorants induced by nitrate (NO_3_^−^) addition and activated sludge recirculation demonstrated moderate efficiency, attributed to the enhanced growth of denitrifying bacteria, which led to a rapid NO_3_^−^ depletion and subsequent recovery of S^2−^.

**Supplementary Information:**

The online version contains supplementary material available at 10.1007/s00449-026-03335-5.

## Introduction

Managing odor emissions in WWTPs is essential for maintaining air quality, ensuring workplace occupational safety, and preserving good community relations, that resulted in multiple odor control strategies developed and implemented in WWTPs to minimize the impact of unpleasant and potentially harmful emissions of different nature. Various chemicals contribute to odor nuisance in WWTPs, including volatile organic compounds, nitrogen-containing compounds, and volatile sulfur compounds (VSCs). The latter comprises hydrogen sulfide (H_2_S) and methyl mercaptan (CH_3_SH), which have been excessively studied over the years due to their malodorous properties, toxicity, and corrosive nature [1]. H_2_S and mercaptans are among the most abundant odoriferous sulphur compounds in WWTPs, producing a range of unpleasant odors (i.e. rotten eggs and cabbage) and exhibiting severe occupational hazards [2, 3]. The hazardous effects on humans of small dosages inhaled of the abovementioned gases have raised public concerns [4], and have been associated with reported short-term health symptoms, including nausea, headaches, coughing, shortness of breath and stress [5].

Several operational zones within WWTPs, often associated with the development of anaerobic or septic conditions, can emit odorous sulfur-containing gases, including influent lines, primary treatment units (e.g. bar screens and primary settlers), and sludge treatment units [6, 7]. In this context, primary treatment facilities are considered responsible for 12–20% of the total odor footprint generated according to multiple studies [6, 8–10]. Design features of primary settlers, such as their large surface area and exposure to wind shear, contribute to widespread odor emissions and intensify the dispersion of malodorants [11, 12]. However, despite the extensive knowledge of sulfur-borne odor mitigation in sewer systems, research on odor mitigation strategies in primary settlers remains scarce. A wide number of research has focused on odour formation and control in sewer systems, primarily using H_2_S as a proxy for malodorants. In this context, Bazemo et al. [12] reported that H_2_S stripping from sewers represents one of the major sources of sulfur-containing odors, accounting for 64% of total H_2_S emissions associated with primary settlers, while the remaining 36% is produced internally via anaerobic sulfate reduction and fermentation process [13]. In contrast, methyl mercaptan emissions are reported to originate mainly within the primary settler, with only 16% being stripped from sewer lines. This highlights the need to address both in-situ production and downstream transport when aiming for effective mitigation of sulfur-based odors in primary settlers [12].

Odor control techniques in WWTPs and sewers aimed at the prevention of foul-smelling substances formation/release, and abatement of odorant compounds that have been emitted [14]. Senatore et al. [15] reported smart process design, buffer zones and masking agents spraying among the most common odor abatement and disguise techniques, along with the implementation of chemical scrubbers, biofiltration, and activated carbon filtration. The prevention of formation and active *in-situ* removal of a specific odorant includes physical-chemical-biological mechanisms, and the inhibition of biological activity with specific bactericidal agents [14]. More specifically, the *in-situ* elimination of sulfur-containing compounds in wastewater (WW) includes a complex chemical and biological oxidation via addition of different reagents (O_2_, NO_3_^−^, MnO_2_, H_2_O_2_, HClO, etc.), precipitation with Fe (II, III) ions, pH elevation, and activated sludge (AS) recirculation. Indeed, the addition of chemical oxidants in sewers demonstrated elimination efficiencies of 70–94% [16–18]. Ferric ions dosage has been traditionally applied as an effective and relatively cheap odor elimination technique with 90–99% elimination efficiencies [19–21]. Similarly, AS recycling has shown promising results in the reduction of sulfureous odors in WWTPs, supporting H_2_S removals of 50–98% [22, 23].

The present study aims at assessing the effectiveness of different odor prevention strategies, including AS and nitrates recycling, the dosing of the precipitating agent FeCl_3_, and the addition of oxidants (H_2_O_2_, NaClO) in mitigating model sulfureous odorants emissions (H_2_S and CH_3_SH) in primary settlers with a constant supply of dissolved sulfide in the inlet works of the settler.

## Materials and methods

### Synthetic wastewater

The pilot scale wastewater treatment plant was fed with a synthetic wastewater (SWW) with a fixed nutrient concentration and a low odour footprint in order to maintain controlled experimental conditions [24]. The SWW contained (mg L^− 1^): glucose 250, meat extract 110, casein peptone 160, NH_2_COH_2_ 30, NaCl 7, CaCl_2_·2H_2_O 4, MgSO_4_ 7H_2_O 2, CuCl_2_·2H_2_O 0.5, K_2_HPO_4_·3H_2_O 112 and NaHCO_3_ 1100. The SWW was freshly prepared three times per week and stored at 4 °C during feeding to avoid degradation.

### Experimental setup

**Fig. 1:**
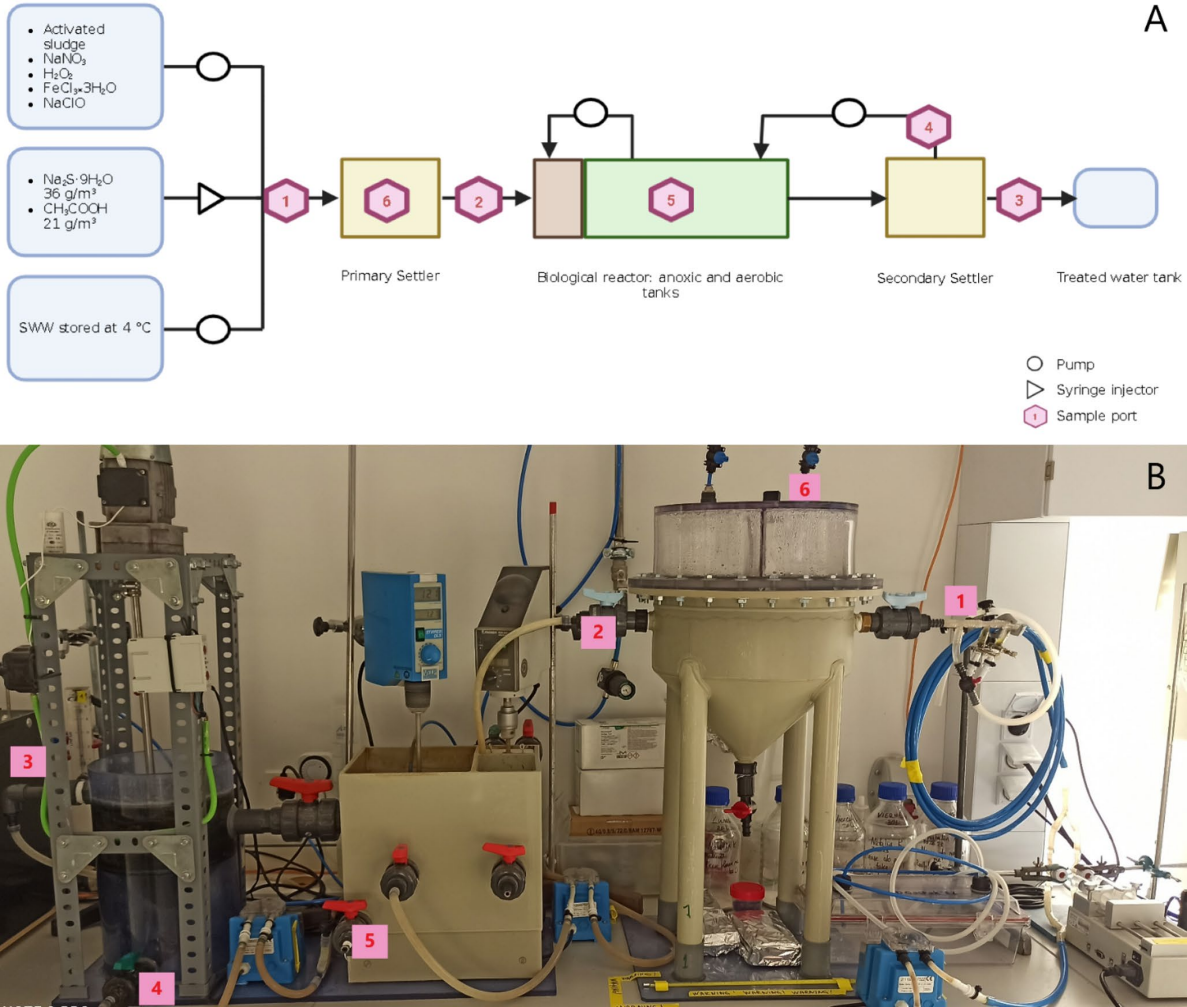
Schematic of the experimental pilot-scale wastewater treatment plant (**A**) and its photograph (**B**): 1 – sampling port of primary settler’s influent wastewater; 2 – liquid sampling port for primary settler effluent; 3 – sampling valve for treated wastewater; 4,5 – AS broth VSS sampling ports: 6 – primary settler headspace sampling port

The pilot scale WWTP consisted of an 8 L primary clarifier (Fig. 1) covered with a gas-tight lid made of transparent polyvinyl chloride (PVC), an 11 L activated sludge (AS) biological reactor and an 8 L secondary settler [25]. The headspace volume of the primary settler was estimated to be 10 L, and the lid was provided with three gas sampling ports. A valve at the bottom of the settler allowed residual sludge collection (0.3 L d^− 1^), which resulted in a sludge retention time (SRT) of 27 days. The 11 L AS reactor was made from polypropylene and consisted of two interconnected chambers for aerobic and anoxic treatment. The AS broth flowed from the aerobic tank into a secondary sludge clarifier made of PVC with a working volume of 8 L. The SWW was fed into the experimental plant using a peristaltic pump (Dosiper C1R) at an influent flowrate (Q) of 0.012 L min^− 1^, resulting in a hydraulic retention time (HRT) of 15.3 h, with a primary settler supporting an HRT of 11 h. The system included a peristaltic recirculation pump (Dosiper C1R) providing the backflow of the mixed liquor from the aerobic tank into the anoxic chamber at 0.024 L min^− 1^. Concurrently, the reverse circulation of the settled sludge from the secondary settler to the anoxic chamber of the biological reactor was performed at a flowrate of 0.012 L min^− 1^ using a peristaltic pump (Dosiper C1R).

A constant supply of sulfur and CH_3_COOH into the inlet SWW was carried out with a syringe pump (Fusion 100, Chemyx, USA) to investigate the fate of VSCs emissions in the primary clarifier. Thus, a sodium sulfide nonahydrate (Na_2_S·9H_2_O) (ThermoScientific, Spain, 98+% ACS) stock solution with a concentration of 124,200 mg L^− 1^, and a stock solution of CH_3_COOH (Cofarcas S.A:, Spain, 99% glacial) at 71,034 mg L^− 1^, were injected into the inlet SWW. Both solutions were injected at a flowrate of 0.21 mL h^− 1^, which resulted in Na_2_S·9H_2_O and CH_3_COOH concentrations of 36 mg L^− 1^ and 21 mg L^− 1^, respectively, to simulate a septic wastewater. A 1.5 m long loop tube was installed prior to the entrance of the primary clarifier to support complete mixing of the SWW and the odorants infused.

###  Experimental phases and sampling

Eleven different experimental phases were carried out from June 2023 until July 2024. The system was operated from November 2022 without any odor treatment mitigation strategy to achieve stable operational conditions. Detailed information of the experimental phases applied, including the type of active reagent, its concentration, and the duration of each experimental phase, is shown in Table 1. Phase I, which served as a baseline for comparison, involved 50 days of operation. Phase II lasted for 38 days and included sodium nitrate (NaNO_3_) injection at a concentration of 15.6 mg N-NO_3_ L^− 1^ into the influent SWW as per Toledo & Muñoz [25]. During phase III, which lasted for 66 days, nitrate dosing at 17.5 mg N-NO_3_ L^− 1^ was carried out with the simultaneous recirculation of settled AS into the influent SWW. For this purpose, 0.28 L of settled AS was drawn daily from the secondary settler and injected into the inlet wastewater stream. Furthermore, H_2_O_2_ dosing at three concentrations of 10, 20 and 50 mg L^− 1^ (ChemREAC, Spain, 33% v/v) was carried out in phases IV, V and VI for 21, 49 and 52 days, respectively [21]. Phases VII and VIII, which lasted for 29 and 34 days, assessed the influence of different FeCl_3_·3H_2_O dosages (40.6 and 81.2 mg L^− 1^) on odorant emissions starting with 1:1 molar ratio in accordance to Nielsen et al. [26]. Following a 10-days stabilization period (phase IX), phases X and XI involved the injection of a commercial sodium hypochlorite (NaClO) solution (Quimicabotin S.L., Spain, 3.46% NaClO) at 91 and 182 mg L^− 1^, respectively, into the influent SWW of the plant [27]. All reagents were stored in 10 L HDPE plastic bottle and introduced into the wastewater stream prior to the looping tube with a peristaltic pump (120 U/DV Watson Marlow, UK).

**Table 1:** Description of the operational conditions evaluated in this study

Phase	Date	Elapsed Time (days)	Chemical reagent	Reagents’ concentration in the WWTP inlet flow (mg L^− 1^)
I	June-July 2023	50	None	None
II	August-September 2023	38	NaNO_3_	15.625 as N-NO_3_
III	September-November 2023	66	NaNO_3_ + AS recirculation	17.500 as N-NO_3_ + AS 450 mL day^− 1^
IV	November 2023	21	H_2_O_2_	10
V	December 2023–January 2024	49	H_2_O_2_	20
VI	February–March 2024	52	H_2_O_2_	50
VII	March–April 2024	29	FeCl_3_·3H_2_O	40.6
VIII	April-May 2024	34	FeCl_3_·3H_2_O	81.2
IX	May 2024	10	None	None
X	June 2024	20	NaClO	91
XI	June-July 2024	20	NaClO	182

Process monitoring was carried out three times per week. The primary settler headspace was sampled and monitored, collecting 0.2 L of gas sample with a 2 L gas-tight Tedlar PVF film sample bag, which was analyzed immediately after collection. Liquid samples for physicochemical analysis were collected at the inlet of the primary settler, at the outlet of the primary settler, and at the outlet of the secondary settler, then filtered (0.2 μm) immediately after a pH level check, diluted accordingly and analyzed later that day.

### Analytical procedures

#### Gas analysis

The headspace gaseous sample was measured with a Residual Gas Analyzer QGA (Hidden Manufacturing, UK) assembled with both Faraday and SEM detectors. This gas analyzer was calibrated using external standards of H_2_S and CH_3_SH (Linde Gas España, Spain) in accordance with Toledo & Muñoz [25].

#### Physicochemical analyses

Total dissolved liquid sulfide concentration (except for elemental sulfur) was measured in accordance with APHA 4500-S^2−^ D methods [28]. The filtered samples were analyzed with a Spectroquant sulfide test kit (Sigma Aldrich, kit #11479) following the instructions of the manufacturer. Spectrophotometric measurements at a wavelength of 665 nm were performed in an absorbance plate reader Spectrostar Nano (BMG LabTech, Germany). The calibration curve with Na_2_S·9H_2_O was prepared in the range of 1–10 mg L^− 1^ of total dissolved sulfides (S^2−^ further in the text). The pH of the samples was measured with a benchtop meter Sension+ pH3 (Hach, USA) calibrated daily with standard solutions. The concentrations of sulfate (SO_4_^2−^) were measured with Waters HPLC system equipped with a 515 HPLC pump, a Waters 717 plus Autosampler, an in-line degasser AF, and a Waters 432 ion conductivity detector (Waters Corporation, USA), according to García et al. [29].

###  Data interpretation

Descriptive statistical analyses were initially conducted to evaluate the central tendencies and variability of the datasets comprising emitted gas concentrations and selected physicochemical parameters. Data reported as mean (standard deviation) [30]. ANOVA one-factor analysis was performed to estimate the difference between datasets with α = 0.05. Removal efficiencies (RE) were calculated according to Eq. (1) and reported with 95% confidence interval (CI):

1 $$\:\begin{array}{c}RE=\left(\frac{{S}_{in}-{S}_{out}}{{S}_{in}}\right)*100\%\end{array}$$

Data analyses were carried out using both Microsoft Excel and IBM SPSS Statistics (29.0.2.0 version, IBM Corp., USA).

## Results and discussion

### Pilot-scale WWTP operation under no-mitigation scenario (baseline phase I)

**Fig. 2:**
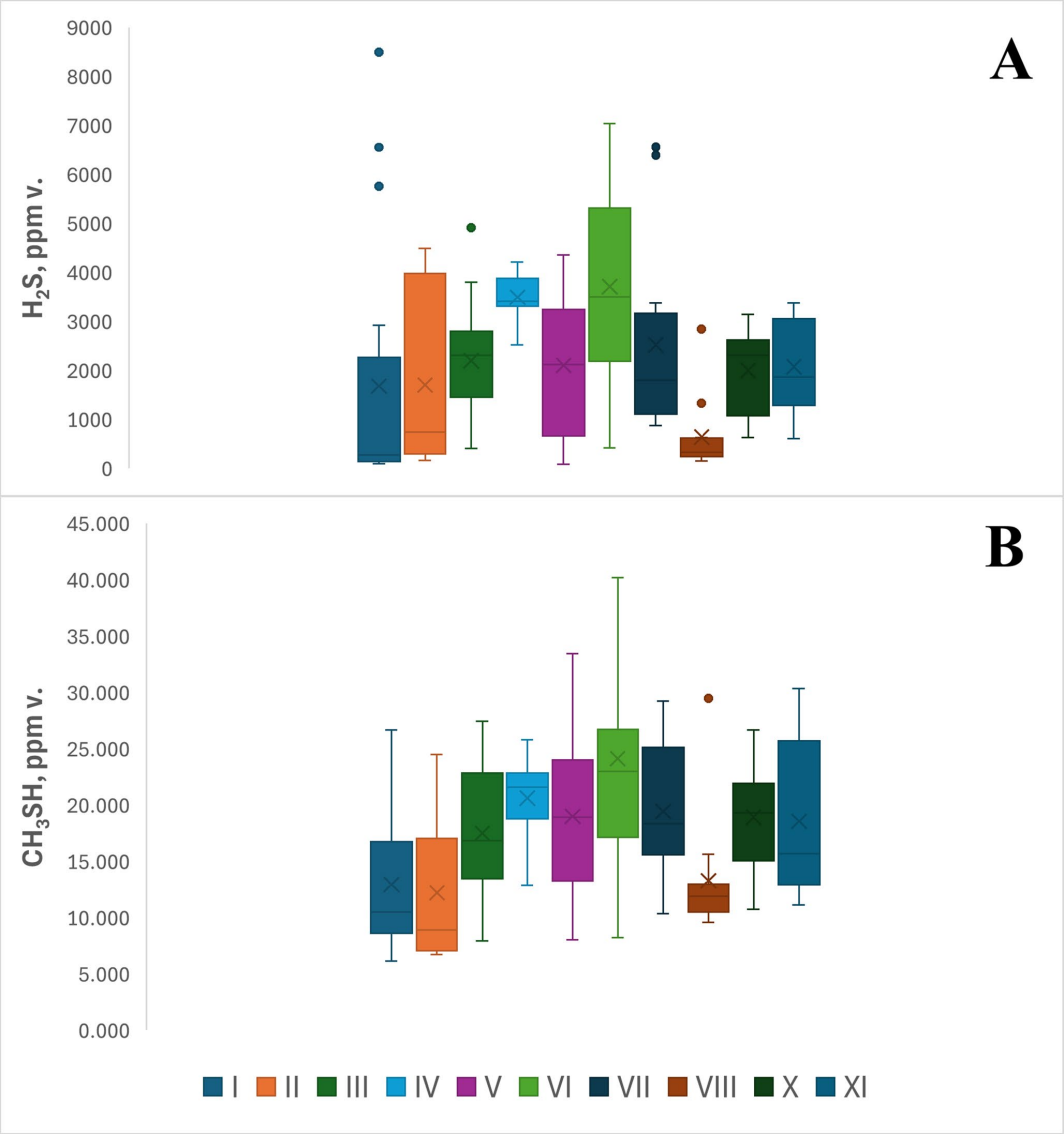
Distribution of concentration data of H_2_S (**A**) and CH_3_SH (**B**) during different operational phases. The number of data points (n) is as follows: I − 18, II – 15, III – 25, IV – 9, V – 16, VI – 18, VII – 11, VIII – 14, X – 9, XI – 9. The difference in H_2_S and CH_3_SH gases concentrations during phases II-XI was later estimated and established significant (*p* < 0.001)

The concentrations of H_2_S and CH_3_SH in the headspace of the settler showed a high degree of variations throughout the entire course of the experiment (Supplementary material, Appendix 1, Fig. S1, S2). The distribution of H_2_S headspace concentrations (Fig. 2) during phase I, ranging from 92.2 ppm v. to 8493 ppm v., demonstrated a substantial variability induced by sulfide stripping from the SWW inflow [12]. The extreme fluctuations in H_2_S concentrations, with a coefficient of variation (CV) equal to 153% (Supplementary material, Appendix 2, Table S1), were consistent with previously reported variability patterns in sulfur-containing gas discharges at WWTPs [31]. Moreover, the H_2_S mean 1685(2580) ppm v. exceeded tenfold the reported concentration of H_2_S emissions for settlers and surpassed hundredfold the highest detected headspace concentration of H_2_S (1.1 ppm v.) under similar conditions by Toledo and Muñoz [8, 12, 25]. The constant injection of Na_2_S and CH_3_COOH into the influent SWW, combined with excessive solids deposition with a subsequent biofilm formation inside the settler for a period of 200 days (November 2022—June 2023) prior to the start of the experiment, could have contributed to the build-up of septicity and a subsequent intensification of H_2_S formation [32]. Likewise, the high CH_3_SH concentrations observed in the headspace of the settler (minimal value of 6.2 ppm v., maximal 26.7 ppm v. with a mean 10.5(1.6) ppm v.) exceeded the reported values on mercaptans emission in WWTP primary settling basins (app. 5.34 ppm v. in summer and 0.21 ppm v. in winter time) and were likely mediated by enhanced microbial activity (Supplementary materials, Appendix 3, Fig.F1) supported by the availability of fermentation products present in the settler [12, 33, 34]. Thus, the complex system of microbial degradation processes backed up with a constant S^2−^ supply, resulted in an active sulfur-borne gases emission during phase I.

For a further and thorough assessment of the sulfur transformation dynamics in the settler, a simplified sulfur species share was estimated for the influent and effluent waters of the settler (Supplementary material, Appendix 1, Fig. S2, S3, Table 2). Only two main sulfur components were considered in this analysis, namely dissolved S^2−^ and SO_4_^2−^ (in mg L^− 1^ of sulfur expressed as S-SO_4_^2−^, S-S^2−^), with the remaining forms likely present in the complex primary settler environment (i.e. elemental sulfur, sulfites, thiosulfates and organically bound sulfur) being excluded.

**Table 2:** Mean concentrations (standard deviation SD) of the dissolved sulfur forms in both influent and effluent of the primary settler (p-value < 0.001 with α = 0.05)

Phase	S-S^2−^influent	S-S^2−^effluent	S-SO_4_^2−^ influent	S-SO_4_^2−^effluent
I	28.8 (9.4)	14.5 (7.2)	3.2 (2.4)	0.74 (1.75)
II	17.0 (11.0)	7.6 (6.7)	1.3 (1.5)	1.7 (1.6)
III	17.1 (12.8)	6.8 (5.6)	3.6 (2.3)	1.3 (2.1)
IV	12.9 (6.2)	6.8 (5.0)	5.6 (1.9)	2.2 (1.4)
V	15.5 (11.4)	6.5 (3.6)	5.3 (2.3)	3.0 (1.9)
VI	14.5 (10.2)	5.0 (4.7)	7.1 (3.2)	3.9 (1.7)
VII	7.1 (6.6)	1.1 (0.5)	3.5 (1.3)	6.8 (3.8)
VIII	0.82 (0.45)	0.7 (0.3)	3.9 (0.7)	5.5 (1.9)
X	13.0 (15.3)	10.2 (10.6)	5.6 (1.6)	6.6 (1.8)
XI	0.51 (0.93)	3.4 (5.0)	7.8 (2.0)	6.2 (1.4)

Table 2 demonstrates that both liquid sulfur forms have demonstrated a high level of dispersion. S-S^2−^ concentrations prevailed in both influent and effluent streams of the primary settler in phase I and averaged 28.8 (9.4) mg L^− 1^ in the influent, with high variations from 12.6 to 44.7 mg L^− 1^ (Supplementary material, Appendix 1, Fig. S2, S3), which matched the constant dosage of 36 mg L^− 1^ of Na_2_S injected. The drop in the settler effluent’s S-S^2−^ concentration down to 14.5 (7.2) mg L^− 1^ further suggested a strong volatilization of a unionized H_2_S into the settler headspace due to pH-depended dissociation [35, 36]. In this context, the depletion of S-SO_4_^2−^ (from 3.2 (2.4) mg L^− 1^ in the influent to 0.74(1.75) mg L^− 1^ in the effluent) supports the assumption that SRB-driven biological reduction of sulfate also took place in the primary settler during phase I resulting in surges in H_2_S and CH_3_SH emissions into the settler headspace [37]. It is important to note that pH levels in both influent and effluent waters of the settler remained neutral in phase I, being 7.4(0.1) (Table 3).

**Table 3:** Average pH levels (with SD) of influent and effluent waters from the settler during phases I-XI

Phase	pH influent	pH effluent
I	7.4 (0.1)	7.4 (0.1)
II	7.5 (0.2)	7.6 (0.2)
III	7.5 (0.1)	7.5 (0.1)
IV	7.5 (0.1)	7.7 (0.2)
V	7.5 (0.1)	7.7 (0.2)
VI	7.4 (0.1)	7.4 (0.1)
VII	7.4 (0.04)	7.4 (0.2)
VIII	7.3 (0.2)	7.2 (0.1)
X	7.2 (0.1)	7.1 (0.04)
XI	7.2 (0.2)	7.0 (0.1)

### Nitrate-induced biological oxidation of sulfureous odorants

Phase II, which involved NO_3_^−^ injections, demonstrated limited effectiveness in mitigating H_2_S emissions due to neutral estimation of RE as well as an increase in both the upper whisker ranges (4486 ppm v. against 2922 ppm v. in phase I) and in the interquartile (IQR) of H_2_S concentration data was observed (Fig. 2A; Table 4). Hence, the latter reached 296 ppm v. – 3983 ppm v., which indicated greater variability and increased emission levels. This lies in contradiction with 90–98% nitrates-induced removal of sulfides reported for sewer systems and wastewater sewage by numerous authors [17, 38, 39]. In addition, the mean of H_2_S gas 1705(1783) ppm v. remained comparable to the baseline period which further suggested that NO_3_^−^ dosage unexpectedly resulted in a limited mitigation of sulfureous gas release.

**Table 4:** Removal efficiencies (%) with 95% confidence interval (CI) calculated for different parameters in phases II-XI

Phase	H_2_S		CH_3_SH		S^2−^ influent	
	RE	CI	RE	CI	RE	CI
II	-0.7	± 51.4	6.0	± 22.4	41.0	± 18.6
III	-28.2	± 24.0	-36.8	± 16.6	40.6	± 17.1
IV	-108	± 28	-60.0	± 14.0	55.2	± 15.0
V	-17.1	± 42.5	-54.4	± 28.7	46.3	± 20.0
VI	-155	± 53	-107	± 50	56.1	± 17.1
VII	72.9	± 13.7	37.6	± 12.4	75.2	± 12.4
VIII	88.5	± 7.3	63.3	± 3.5	97.1	± 0.9
X	6.7	± 28.4	-44.6	± 26.3	54.8	± 32.6
XI	39.7	± 19.2	-21.1	± 30.8	98.2	± 1.7

The results show a drop in S-S^2−^ concentration to 17.0(11.0) mg L^− 1^ and 7.3(6.7) mg L^− 1^ in the influent and effluent streams, respectively, compared to the baseline period, which could indicate that biological sulfide oxidation proceeded according to Eqs. (2) and (3) [40].

2 $$\:\begin{array}{c}5{S}^{2-}+2{NO}_{3}^{-}+12{H}^{+}=5{S}^{0}+{N}_{2}+6{H}_{2}O\end{array}$$

3 $$\:\begin{array}{c}5{S}^{0}+6{NO}_{3}^{-}+2{H}_{2}O=5{SO}_{4}^{2-}+3{N}_{2}+4{H}^{+}\end{array}$$

However, there is no substantial evidence of elemental sulfur oxidation, as S^0^ was not measured during the experimental phases. Moreover, the decline in sulfate levels in the influent water, along with an insignificant rise in effluent pH to 7.6(0.2) in the primary settler, may indirectly indicate slower oxidative transformation rates of sulfur (≈ 15%), as reported by Jiang et al. [40]. At the same time, the observed increase in VSC emissions from the primary settler could be attributed to enhanced activity of SRB, supported by higher S-SO_4_^2−^ concentrations in the effluent, potentially resulting from a secondary step of nitrate-induced oxidation [40–42]. Finally, CH_3_SH emissions, with a mean of 12.2(6.0) ppm v. (Fig. 2B), remained unchanged compared to the baseline period, indicating the limited impact of NO_3_^−^-induced biological oxidation on mercaptan emissions [43].

### Nitrate injection coupled with AS recirculation to enhance the oxidation of sulfureous odorants

The continuous nitrate supply combined with AS recycling implemented in phase III did not mitigate sulfur-containing gas emissions as the REs calculated remained negative and mean concentrations (Fig. 2A, B) of both of H_2_S and CH_3_SH increased (2199(1043) ppm v. and 17.5(5.5) ppm v., respectively) (Table 4). This contradicts the latest research by Toledo and Muñoz [25] that achieved a 95% H_2_S removal efficiency (RE) under similar conditions (3.6% v/v AS ratio combined with a nitrate dosing 10–20 mg NL^− 1^). Moreover, Zhang et al. [44] reported a substantial mitigation effect in dissolved sulfide concentration (99% removal of S^2−^) in sewage with an increased AS ratio (16% v/v). In the latter study, however, sulfide elimination could be attributed to the presence of Fe forms in the AS, as evidenced by the lower sulfide RE (53%) observed in the same study when AS with a low Fe content was recycled [44]. However, the larger extent in the development of the biofilm inside the primary settler might explain the limited efficiency of the combined nitrate and AS injection in current study.

At the same time, Pang et al. [45] demonstrated that biodegradation was a predominant mechanism of sulfide removal in AS recirculation systems under an increased dissolved oxygen (DO) concentration > 6.5 mg L^− 1^, whilst adsorption on sludge particles without further S^2−^ bioconversion occurred at low DO levels (< 0.5 mg L^− 1^). Therefore, under the operational conditions implemented in the current work (DO in the primary settler was approx. 0.40 mg L^− 1^ as per Sander [46]), the recirculated AS could not exhibit sufficient biological oxidation capacity to support a robust odorant mitigation effect. However, a drop of S-S^2−^ remained in the influent of the primary settler resulting in a median 17.1(12.8) mg L^− 1^ (Table 2), that suggested a continuous process of nitrate-based oxidation of sulfides in the inlet waters prior to the entrance to the settler probably catalyzed by the combined share of NO_3_^−^ ions brought with AS waters (1.840 N-NO_3_^−^ mg L^− 1^) [36]. However, a low sulfide RE (40.6 ± 17.1%) in the settler influent water in phase III, combined with the intensification of sulfureous gases emissions, could indicate that a pattern of two-step NO_3_^−^ oxidation similar to the one detected in phase II has occurred in phase III as well (Table 4).

###  Chemical oxidation of sulfureous odorants with H_2_O_2_

Chemical oxidation of dissolved S^2−^ and gaseous H_2_S with H_2_O_2_ was proven to be efficient (87–99% removals) by various researchers in sewers and in sulfide-containing sludges [21, 47–50]. However, the current research showed a limited elimination capacity of H_2_S and CH_3_SH in the primary settler headspace due to negative REs estimated and elevated levels of both H_2_S (3494(487) ppm v., 2105(1388) ppm v., 3710(1937) ppm v.) and CH_3_SH (20.6(3.69) ppm v., 19.0(7.5) ppm v. and 24.1(12.6) ppm v.) in phases IV, V and VI, respectively (Supplementary material, Appendix 2, Table S1, S2). Interestingly, phase IV (10 mg L^− 1^ injection of H_2_O_2_) was characterized by a relatively narrow IQR for H_2_S (3409 ppm v. – 3879 ppm v.) and for CH_3_SH (18.8 ppm v. – 22.8 ppm v.), and the lowest CV of gaseous emissions was observed (13.8% of H_2_S and 17.7% of CH_3_SH), which indicated a reduced variability and the lowest fluctuation in emissions of both gases. Moreover, the spiked S-SO_4_^2−^ concentrations in the range of 5.3–7.1 mg L^− 1^ in the settler influent in phases IV-VI indicate the oxidation processes present in the inlet WWs of the system according to Eq. (4) [47].

4 $$\:\begin{array}{c}{S}^{2-}+4{H}_{2}{O}_{2}={SO}_{4}^{2-}+4{H}_{2}O\end{array}$$

Supporting this, S-S^2−^ level in the influent SWW remained decreased (12.9(6.2) mg L^− 1^, 15.5(11.4) mg L^− 1^ and 14.5(10.2) mg L^− 1^ in phases IV, V and VI, respectively) comparing to the baseline phase I, which further confirmed the oxidation potential of H_2_O_2_, although insufficient to establish a positive sulfide removal rate. Additionally, the molar ratio of O^2−^:S^2−^ in this experiment gradually increased from 2 to 10 moles of O^2−^ per 1 mol of dissolved sulfide, which exceeded the tested 1–4:1 ratio estimated for the full oxidation of S^2−^ with peroxide by various researchers [47, 51, 52]. In this regard, Hoffman M [47]. demonstrated *in vitro* that at neutral pH the optimal ratio of peroxide to sulfide is 2:1, as higher dosages of the oxidant led to a higher percentage of sulfate formation and a potential shift in the oxidation products from elemental sulfur toward sulfates. This suggest that, under the complex conditions of primary settlers (where anaerobic conditions prevail at neutral pH) chemical sulfide oxidation will primarily produce SO_4_^2−^ when higher doses of peroxide are applied, which, in turn, could enhance H_2_S emissions due to extensive SRB activity. Therefore, to minimize VSC emissions in settlers, the use of hydrogen peroxide appears unsuitable, as its addition could indirectly promote H_2_S and CH_3_SH emissions through the sulfate-producing oxidation pathway.

### Sulfureous odorants mitigation via ferric chloride precipitation

Phases VII and VIII exhibited a continuous and pronounced decline in H_2_S and CH_3_SH headspace concentrations, confirming this method as the most effective odor mitigation strategy. H_2_S REs reached 72.9 ± 13.7% in phase VII and 88.5 ± 7.3% in phase VIII, while REs of CH_3_SH accounted for 37.6 ± 12.4% and 63.3 ± 3.5%, respectively (Table 4). Our findings align with multiple literature studies that reported high REs (70%-99%) of sulfur compounds using iron salts in WWTP and sewers [19, 53, 54]. Moreover, the median H_2_S and CH_3_SH concentration in phase VIII were the lowest observed among the tested treatment phases (645(763) ppm v. and 13.3(5.4) ppm v., respectively), which strongly support the effectiveness of an increased FeCl_3_ dosage in mitigation of sulfur-containing gas emissions. Furthermore, the dissolved S^2−^ removal was achieved concurrently in the settler as both influent and effluent streams has demonstrated a drop of S-S^2−^ concentrations to 0.82(0.45) mg L^− 1^ and 0.69(0.34) mg L^− 1^ in phase VIII, which corresponded to RE 97.1 ± 0.9% (Table 3). This could be explained by a rapid bounding of the injected S^2−^ with Fe (III) inside and prior to the entrance of the settler [20, 55]. Interestingly, S-SO_4_^2−^ concentrations in the settler influent remained stable at 3.5(1.3) mg L^− 1^ and 3.9(0.7) mg L^− 1^ during phases VII and VIII, respectively, which rule out the occurrence of any sulfur oxidation mechanisms. At the same time, the elevated levels of S-SO_4_^2−^ in the settler effluent (6.8(3.8) mg L^− 1^ and 5.5(1.9) mg L^− 1^ in phases VII and VIII) suggest a halt in biological sulfate reduction within the settler, probably due to the inhibitory effects of the elevated metal sulfide concentrations [56]. In our study, the removal efficiencies of H_2_S gas were significantly higher than those of CH_3_SH, which can be attributed to the complex and interchangeable fate of methyl mercaptan emissions as a byproduct of both sulfur methylation and amino acid degradation [57]. Despite no report was found in literature investigating the effect of Fe ions on CH_3_SH removal, Yongchao et al. [58] described REs of 99% for both H_2_S and CH_3_SH under Fe-C dosing in sewers. These results align with the positive effect of Fe (III) injections on CH_3_SH mitigation (63.3 ± 3.5% removals in phase VIII), although the mechanisms of methyl mercaptan elimination remain unknown to the best of our knowledge.

In this context, Kulandaivelu et al. [19] reported that a Fe (III): S^2−^ molar ratio of 2.8:1 and 3.4:1 supported REs of both dissolved sulfides and gaseous H_2_S > 90% under treatment with 30% FeCl_3_·3H_2_O dosage at the inlet syphon of a WWTP. Moreover, Ahmed et al. [59] proved Fe (III): S^2−^ molar ratio of 2:1 to be effective with 89% sulfide removals. These correspond with the optimal performance achieved in phase VIII at a Fe (III): S^2−^ molar ratio of 2:1. On the other hand, the tested ratio 1:1 in phase VII exhibited a limited capacity for sulfureous gas and dissolved sulfide mitigation. Likewise, Nielsen [60] reported a limited effectiveness of sulfide precipitation at molar ratios of 1.0 mol in a lab-scale sewer, which could be explained by the change in iron availability by ligands in WW. Additionally, a US EPA report identified iron dosage at ratios > 10:1 Fe (II) or (III): S as the most effective strategy for sulfide control [61]. Thus, a successful removal of S^2−^ and VSC emitted requires ratios ≥ 2:1 Fe (III) /S^2−^.

### Chemical oxidation of sulfureous odorants via hypochlorite addition

Following a 10-day interruption in treatment during phase IX, a notable increase in sulfur gas emissions was observed in phase X with concentrations of 1998(868) ppm v. for H_2_S and 18.9(4.9) ppm v. for CH_3_SH. This indicated a rapid rebound of microbial sulfate reduction and organic matter degradation in the absence of odor mitigation procedures. Interestingly, the concentrations of H_2_S and CH_3_SH did not significantly decline, reaching 2078(985) ppm v. and 18.6(7.0) ppm v., respectively in phase XI, demonstrating the limited effectiveness of the increased NaClO dosage on the control of sulfur-containing gaseous emissions. Previous studies reported removals of 90–99% for both sulfide and H_2_S when dosing NaClO in sewers and WWs due to the strong oxidation capacity of the reagent combined with its bactericidal properties [21, 62–64]. These findings could partially correlate with the results of the current investigation, since during phase XI, the removal of dissolved S^2−^ reached 98.2 ± 1.7% in the settler influent with an average of 0.51(0.93) mg L^− 1^ (Table 4), confirming the convincing performance of the increased dosage of the oxidizing agent on dissolved sulfide oxidation in the influent SWW. The abovementioned results suggest that the optimal dosage ratio of Cl^−^ to S^2−^ necessary for the full oxidation of dissolved sulfide was 16:1 in phase XI, which exceeded two times the stoichiometric value reported for WWTPs by Waltrip & Snyder [21]. Furthermore, the observed increase in S-SO_4_^2−^ concentrations (Table 3) in the influent WWs with 5.6(1.6) mg L^− 1^ in phase X and 7.8(2.0) mg L^− 1^ in phase XI, strongly supports NaClO-induced oxidation yielding sulfates that took place in the influent waters [52]. At the same time, a rise in sulfate level in the effluent waters of the primary settler in phase X reaching 6.6(1.8) mg L^− 1^ and 6.2(1.4) mg L^− 1^ in phase XI along with a drop in pH of both streams (Table 3) could indicate the anticipated presence of sulfur as one of the products of oxidation and further suggest an overall exclusion of some portion of sulfur from the settler cycle due to elemental sulfur’s low solubility since there was no obvious increase in gaseous discharge [52, 65, 66]. The abovementioned compared absence of change in gaseous H_2_S and CH_3_SH concentrations detected in phase XI could further suggest the change in sulfur/sulfate ratio of hypochlorite oxidation products as well as the reduced bactericidal properties of the reagent. Therefore, it could be presumed that the use of NaClO as dissolved sulfide oxidant in the set-up influent waters could prove useful, though under septic conditions inside the settler with a developed biofilm, the reagent`s dosages utilized demonstrated low efficiency in both SRB inhibition and sulfureous gases mitigation.

## Conclusions

The tested methods for mitigating odorous sulfur compounds exhibited varying efficiencies in settlers compared to those observed in sewers systems. Ferric chloride dosing was shown to be the most effective method for mitigating H_2_S and CH_3_SH emissions from primary settlers during municipal wastewater treatment, due to its dual capacity to both bind dissolved sulfide and inhibit SRB activity. A molar ratio of 2:1 Fe (III) to S^2−^ showed removals of 88.5 ± 7.3% and 63.3 ± 3.5% for H_2_S and CH_3_SH, respectively. Moreover, the elimination of dissolved S^2−^ with Fe (III) ions was found to be an effective technique, as 97.1 ± 0.9% of the injected sulfide was precipitated with ferric chloride dosage. Chemical oxidation using NaClO was also found to be an effective method for dissolved sulfide removal, with an estimated S^2−^ RE of 98.2 ± 1.7%. However, mitigation of gaseous H_2_S and CH_3_SH with NaClO was limited, likely due to the insufficient bactericidal effect of the applied dosages. In contrast, nitrate dosing, AS recirculation combined with nitrate addition, and H_2_O_2_ dosage showed low effectiveness in controlling both H_2_S and CH_3_SH emissions, as well as S^2−^ levels. These methods presumably target primarily one form of sulfur present in the settler, namely, dissolved sulfide, which originates mostly in the upstream sections of the WWTPs. To achieve effective VSC mitigation in primary settlers, it is advisable to target both sources of sulfur odor: the dissolved S^2−^, which can be directly emitted via pH-driven equilibrium, and the H_2_S produced biologically in situ from sulfate reduction within the settler.

## Supplementary Information

Below is the link to the electronic supplementary material.

Supplementary Material 1

## Data Availability

All data supporting the findings of this study are available within the paper and its Supplementary materials.

## Abbreviations

AS: Activated sludge
DO: Dissolved oxygen
CI: Confidence interval
PVC: Polyvinyl chloride
RE: Removal efficiency
S^2−^: Total dissolved sulfides, sulfides as a mixture of dissolved H_2_S, HS^−^ and S^2^
SRB: Sulfate reducing bacteria
SWW: Synthetic wastewater
VSC: Volatile sulfur compounds (H_2_S, CH_3_SH)
WWTP: Wastewater treatment plant
WW: Wastewater

## References

[1] Jiang G, Sun J, Sharma KR, Yuan Z (2015) Corrosion and odor management in sewer systems, *Curr. Opin. Biotechnol.*, vol. 33, pp. 192–197, Jun. 10.1016/J.COPBIO.2015.03.007 10.1016/j.copbio.2015.03.007 25827114

[2] Lewkowska P, Cieślik B, Dymerski T, Konieczka P, Namieśnik J (Nov. 2016) Characteristics of odors emitted from municipal wastewater treatment plant and methods for their identification and deodorization techniques. Environ Res 151:573–586. 10.1016/J.ENVRES.2016.08.030 10.1016/j.envres.2016.08.030 27591529

[3] Shi X et al (2022) Evaluating the oxidation inhibition of sulfide in urban sewers using a novel quantitative method. Chemosphere 296:133958. 10.1016/j.chemosphere.2022.133958 35176294 10.1016/j.chemosphere.2022.133958

[4] Piccardo MT, Geretto M, Pulliero A, Izzotti A (Mar. 2022) Odor emissions: A public health concern for health risk perception. Environ Res 204:112121. 10.1016/J.ENVRES.2021.112121 10.1016/j.envres.2021.112121 34571035

[5] Schiffman SS et al (2000) Potential Health Effects of Odor from Animal Operations, Wastewater Treatment, and Recycling of Byproducts. J Agromedicine 7(1):7–81. 10.1300/J096v07n01_02 19785232

[6] Czarnota J, Masłoń A, Pajura R (2023) Wastewater Treatment Plants as a Source of Malodorous Substances Hazardous to Health, Including a Case Study from Poland. Int J Environ Res Public Health 20(7). 10.3390/ijerph20075379 10.3390/ijerph20075379 PMC10093992 37047993

[7] Stellacci P, Liberti L, Notarnicola M, Haas CN (2010) Hygienic sustainability of site location of wastewater treatment plants: A case study. I. Estimating odour emission impact, *Desalination*, vol. 253, no. 1–3, pp. 51–56, Apr. 10.1016/J.DESAL.2009.11.034

[8] Karageorgos P, Latos M, Kotsifaki C, Lazaridis M, Kalogerakis N (2010) Treatment of unpleasant odors in municipal wastewater treatment plants. Water Sci Technol 61(10):2635–2644. 10.2166/wst.2010.211 20453338 10.2166/wst.2010.211

[9] Senatore V et al (2021) Sustainable Odour and Greenhouse Gas Emissions Control in Wastewater Treatment Plant by Advanced Biotechnology-based System. Chem Eng Trans 85:25–30. 10.3303/CET2185005

[10] Zarra T, Naddeo V, Belgiorno V, Reiser M, Kranert M (2008) Odour monitoring of small wastewater treatment plant located in sensitive environment. Water Sci Technol 58(1):89–94. 10.2166/wst.2008.330 18653941 10.2166/wst.2008.330

[11] Prata AA, Santos JM, Timchenko V, Stuetz RM (2018) A critical review on liquid-gas mass transfer models for estimating gaseous emissions from passive liquid surfaces in wastewater treatment plants, *Water Res.*, vol. 130, pp. 388–406, Mar. 10.1016/J.WATRES.2017.12.001 10.1016/j.watres.2017.12.001 29258050

[12] Bazemo U et al (2021) Investigating the dynamics of volatile sulfur compound emission from primary systems at a water resource recovery facility. Water Environ Res 93(2):316–327. 10.1002/wer.1417 32706455 10.1002/wer.1417

[13] Stuetz R, Frechen FB (2005) *Odours in Wastewater Treatment - Measurement, Modelling and Control*. IWA Publishing, [Online]. Available: https://iwaponline.com/ebooks/book/29/Odours-in-Wastewater-Treatment-Measurement

[14] Talaiekhozani A, Bagheri M, Goli A, Talaei Khoozani MR (2016) An overview of principles of odor production, emission, and control methods in wastewater collection and treatment systems. J Environ Manage 170:186–206. 10.1016/j.jenvman.2016.01.021 26829452 10.1016/j.jenvman.2016.01.021

[15] Senatore V et al (2021) Dec., Full-Scale Odor Abatement Technologies in Wastewater Treatment Plants (WWTPs): A Review, *Water 2021, Vol. 13, Page 3503*, vol. 13, no. 24, p. 3503. 10.3390/W13243503

[16] Bentzen G et al (1995) Controlled dosing of nitrate for prevention of H2S in a sewer network and the effects on the subsequent treatment processes. Water Sci Technol 31(7):293–302. 10.2166/wst.1995.0245

[17] García J, De Lomas A, Corzo JM, Gonzalez JA, Andrades E, Iglesias, Montero MJ (2006) Nitrate promotes biological oxidation of sulfide in wastewaters: Experiment at plant-scale, *Biotechnol. Bioeng.*, vol. 93, no. 4, pp. 801–811, Mar. 10.1002/BIT.20768 10.1002/bit.20768 16255035

[18] Mohanakrishnan J et al (2009) Impact of nitrate addition on biofilm properties and activities in rising main sewers. Water Res 43:4225–4237. 10.1016/j.watres.2009.06.021 19577270 10.1016/j.watres.2009.06.021

[19] Kulandaivelu J et al (2020) Full-scale investigation of ferrous dosing in sewers and a wastewater treatment plant for multiple benefits. Chemosphere 250:126221. 10.1016/j.chemosphere.2020.126221 32114337 10.1016/j.chemosphere.2020.126221

[20] Padival NA, Kimbell WA, Redner JA (1995) Use of Iron Salts to Control Dissolved Sulfide in Trunk Sewers. J Environ Eng 121(11):824–829

[21] Waltrip GD, Snyder EG (1985) Elimination of Odor at Six Major Wastewater Treatment Plants, *J. Water Pollut. Control Fed.*, vol. 57, no. 10, pp. 1027–1032, [Online]. Available: https://www.jstor.org/stable/25042774

[22] Harrison K, Davis J, Seymour Z, Johnson L, Dalton D Control of Odor Emissions from Aeration Basins: The Palm Beach Experience, in *Odors and Air Pollutants Conference* (2008), Water Environment Federation, 2008, pp. 893–908. [Online]. Available:, Water Environment Federation, 2008, pp. 893–908. [Online]. Available: https://www.accesswater.org/publications/proceedings/-295625/control-of-odor-emissions-from-aeration-basins--the-palm-beach-experience

[23] Schmidt D, Karleskint J, Porter J, Parker D, Hoskins K Mixing RAS with raw sewage to reduce odor emissions while avoiding adverse impacts to processes, in *Odors and Air Pollutants Conference* (2014), Water Environment Federation, 2014. [Online]. Available:, Water Environment Federation, 2014. [Online]. Available: https://www.accesswater.org/publications/proceedings/-282665/mixing-ras-with-raw-sewage-to-reduce-odor-emissions-while-avoiding-adverse-impacts-to-processes

[24] O’Flaherty E, Gray NF (2013) A comparative analysis of the characteristics of a range of real and synthetic wastewaters. Environ Sci Pollut Res 20(12):8813–8830. 10.1007/s11356-013-1863-y 10.1007/s11356-013-1863-y 23740303

[25] Toledo M, Muñoz R (Oct. 2023) Odour prevention strategies in wastewater treatment plants: A pilot scale study of activated sludge recycling and oxidized nitrogen recycling. J Environ Chem Eng 11(5):110366. 10.1016/J.JECE.2023.110366

[26] Nielsen AH, Lens P, Vollertsen J, Hvitved-Jacobsen T (Jul. 2005) Sulfide–iron interactions in domestic wastewater from a gravity sewer. Water Res 39(12):2747–2755. 10.1016/J.WATRES.2005.04.048 10.1016/j.watres.2005.04.048 15978649

[27] Huang Y, Liu Z, Guo Y, Lin Q, Liao X, Qi H (2020) A comparative study on sulfide removal by HClO and KMnO 4 in drinking water. Environ Sci (Camb) 6(10):2871–2880. 10.1039/D0EW00629G

[28] Lipps EB, Braun-Howland, Baxter TE (2023) *Standard Methods for the Examination of Water and Wastewater. American Public Health Association, American Water Works Association, Water Environment Federation APHA*, 24th ed. Washington DC: APHA Press, [Online]. Available: https://www.standardmethods.org/

[29] García D, Alcántara C, Blanco S, Pérez R, Bolado S, Muñoz R (2017) Enhanced carbon, nitrogen and phosphorus removal from domestic wastewater in a novel anoxic-aerobic photobioreactor coupled with biogas upgrading. Chem Eng J 313:424–434. 10.1016/j.cej.2016.12.054

[30] Lang TA, Altman DG (Jan. 2015) Basic statistical reporting for articles published in Biomedical Journals: The ‘Statistical Analyses and Methods in the Published Literature’ or the SAMPL Guidelines. Int J Nurs Stud 52(1):5–9. 10.1016/J.IJNURSTU.2014.09.006 10.1016/j.ijnurstu.2014.09.006 25441757

[31] Lafita C, Penya-Roja JM, Sempere F, Waalkens A, Gabaldón C (Jun. 2012) Hydrogen sulfide and odor removal by field-scale biotrickling filters: Influence of seasonal variations of load and temperature. J Environ Sci Health Tox Hazard Subst Environ Eng 47(7):970–978 2012.667302;REQUESTEDJOURNAL:JOURNAL:LESA20;WGROUP:STRING:PUBLICATION 10.1080/10934529.2012.667302 22486666

[32] Boon AG, Vincent AJ (2003) Odour generation and control, *Handbook of Water and Wastewater Microbiology*, pp. 545–557, Jan. 10.1016/B978-012470100-7/50034-0

[33] Faris AM et al (2022) Fate and emission of methyl mercaptan in a full-scale MBBR process by TOXCHEM simulation. J Water Clim Change 13(6):2386–2398. 10.2166/wcc.2022.438

[34] Jeon E-C, Son H-K, Sa J-H (2009) Emission Characteristics and Factors of Selected Odorous Compounds at a Wastewater Treatment Plant. Sens (Basel) 9(1):311–326. 10.3390/s90100311 10.3390/s90100311 PMC3280747 22389601

[35] Wu S, Kuschk P, Wiessner A, Müller J, Saad RAB, Dong R (2013) Sulphur transformations in constructed wetlands for wastewater treatment: A review, *Ecol. Eng.*, vol. 52, pp. 278–289, Mar. 10.1016/J.ECOLENG.2012.11.003

[36] Yang W, Vollertsen J, Hvitved-Jacobsen T (2005) Anoxic sulfide oxidation in wastewater of sewer networks. Water Sci Technol 52(3):191–199. 10.2166/wst.2005.0076 16206859

[37] Barton LL, Hamilton WA (2007) *Sulphate-Reducing Bacteria: Environmental and Engineered Systems*. Cambridge: Cambridge University Press, [Online]. Available: https://www.cambridge.org/core/books/sulphatereducing-bacteria/1DC53B7E35A5FAE34E2536D03B1E5E98

[38] Auguet O, Pijuan M, Guasch-Balcells H, Borrego CM, Gutierrez O (Jan. 2015) Implications of Downstream Nitrate Dosage in anaerobic sewers to control sulfide and methane emissions. Water Res 68:522–532. 10.1016/J.WATRES.2014.09.034 10.1016/j.watres.2014.09.034 25462758

[39] Can-Dogan E, Turker M, Dagasan L, Arslan A (2010) Sulfide removal from industrial wastewaters by lithotrophic denitrification using nitrate as an electron acceptor, *Water Science and Technology*, vol. 62, no. 10, pp. 2286–2293, Nov. 10.2166/WST.2010.545 10.2166/wst.2010.545 21076214

[40] Jiang G, Sharma KR, Guisasola A, Keller J, Yuan Z (2009) Sulfur transformation in rising main sewers receiving nitrate dosage, *Water Res.*, vol. 43, no. 17, pp. 4430–4440, Sep. 10.1016/J.WATRES.2009.07.001 10.1016/j.watres.2009.07.001 19625067

[41] Liang Z-S, Zhang L, Wu D, Chen G-H, Jiang F (2019) Systematic evaluation of a dynamic sewer process model for prediction of odor formation and mitigation in large-scale pressurized sewers in Hong Kong. Water Res 154:94–103. 10.1016/j.watres.2019.01.033 30776618 10.1016/j.watres.2019.01.033

[42] Liu G, Wang J (2015) Modeling effects of DO and SRT on activated sludge decay and production. Water Res 80:169–178. 10.1016/j.watres.2015.04.042 26001822 10.1016/j.watres.2015.04.042

[43] Tanimoto Y, Bak F (1994) Anaerobic degradation of methylmercaptan and dimethyl sulfide by newly isolated thermophilic sulfate-reducing bacteria. Appl Environ Microbiol 60(7):2450–2455. 10.1128/AEM.60.7.2450-24551994;CTYPE:STRING:JOURNAL 8074524 10.1128/aem.60.7.2450-2455.1994 PMC201669

[44] Zhang L et al (2011) Addition of an aerated iron-rich waste-activated sludge to control the soluble sulphide concentration in sewage. Water Environ J 25(1):106–115. 10.1111/j.1747-6593.2009.00198.x

[45] Pang BW, Jiang CH, Yeung M, Ouyang Y, Xi J (Feb. 2017) Removal of dissolved sulfides in aqueous solution by activated sludge: mechanism and characteristics. J Hazard Mater 324:732–738. 10.1016/J.JHAZMAT.2016.11.048 10.1016/j.jhazmat.2016.11.048 27894757

[46] Sander R (2023) Compilation of Henry’s law constants (version 5.0.0) for water as solvent. Atmos Chem Phys 23:10901–12440. 10.5194/acp-23-10901-2023

[47] Hoffmann MR (1977) Kinetics and Mechanism of Oxidation of Hydrogen Sulfide by Hydrogen Peroxide in Acidic Solution, *Environ. Sci. Technol.*, vol. 11, no. 1, pp. 61–66, Jan. 10.1021/ES60124A004

[48] Ksibi M (2006) Chemical oxidation with hydrogen peroxide for domestic wastewater treatment. Chem Eng J 119(2):161–165. 10.1016/j.cej.2006.03.022

[49] Zhang J, Liao Y, Wang Q, Wang C, Yu J (Feb. 2021) Degradation of odorous sulfide compounds by different oxidation processes in drinking water: Performance, reaction kinetics and mechanism. Water Res 189:116643. 10.1016/J.WATRES.2020.116643 10.1016/j.watres.2020.116643 33246216

[50] Yin R, Peng J, Sun J, Li C, Xia D, Shang C (2021) Simultaneous removal of hydrogen sulfide, phosphate and emerging organic contaminants, and improvement of sludge dewaterability by oxidant dosing in sulfide-iron-laden sludge. Water Res 203:117557. 10.1016/j.watres.2021.117557 34418644 10.1016/j.watres.2021.117557

[51] Guzman KJ (2007) Kinetics of Hydrogen Sulfide Oxidation in Sanitary Sewer Systems, Accessed: Sep. 09, 2025. [Online]. Available: https://scholarworks.uno.edu/td/551

[52] Cadena F, Peters RW (1988) Evaluation of Chemical Oxidizers for Hydrogen Sulfide Control, *J. Water Pollut. Control Fed.*, vol. 60, no. 7, pp. 1259–1263, [Online]. Available: https://www.jstor.org/stable/25043633

[53] Gutierrez O, Park D, Sharma KR, Yuan Z (2010) Iron salts dosage for sulfide control in sewers induces chemical phosphorus removal during wastewater treatment. Water Res 44(11):3467–3475. 10.1016/j.watres.2010.03.023 20434190 10.1016/j.watres.2010.03.023

[54] Zhang L, Keller J, Yuan Z (2009) Inhibition of sulfate-reducing and methanogenic activities of anaerobic sewer biofilms by ferric iron dosing. Water Res 43:4123–4132. 10.1016/j.watres.2009.06.013 19576610 10.1016/j.watres.2009.06.013

[55] Jameel P (1989) The Use of Ferrous Chloride to Control Dissolved Sulfides in Interceptor Sewers, *J. Water Pollut. Control Fed.*, vol. 61, no. 2, pp. 230–236, [Online]. Available: https://www.jstor.org/stable/25046917

[56] Utgikar VP, Harmon SM, Chaudhary N, Tabak HH, Govind R, Haines JR (Jan. 2002) Inhibition of sulfate-reducing bacteria by metal sulfide formation in bioremediation of acid mine drainage. Environ Toxicol 17(1):40–48. 10.1002/TOX.10031 10.1002/tox.10031 11847973

[57] Van Leerdam RC, De Bok FAM, Lomans BP, Stams AJM, Lens PNL, Janssen AJH (2006) Volatile organic sulfur compounds in anaerobic sludge and sediments: Biodegradation and toxicity, *Environ. Toxicol. Chem.*, vol. 25, no. 12, pp. 3101–3109, Dec. 10.1897/06-106R.1 10.1897/06-106r.1 17220077

[58] Yongchao Z, Lei T, Wenming Z, Yiping Z, Lei F, Tuqiao Z (Jan. 2023) Iron carbon particle dosing for odor control in sewers: Laboratory tests. Environ Res 216:114476. 10.1016/J.ENVRES.2022.114476 10.1016/j.envres.2022.114476 36202246

[59] Ahmed M, Saup CM, Wilkins MJ, Lin LS (Apr. 2020) Continuous ferric iron-dosed anaerobic wastewater treatment: Treatment performance, sludge characteristics, and microbial composition. J Environ Chem Eng 8(2):103537. 10.1016/J.JECE.2019.103537

[60] Nielsen AH, Hvitved-Jacobsen T, Vollertsen J (2008) Effects of pH and Iron Concentrations on Sulfide Precipitation in Wastewater Collection Systems. Water Environ Res 80(4):380–384. 10.2175/106143007X221328 18536490 10.2175/106143007x221328

[61] US EPA (1985) Odor and Corrosion Control in Sanitary Sewerage Systems and Treatment Plants, Cincinnati, 1985. Accessed: Sep. 01, 2025. https://cfpub.epa.gov/si/si_public_record_report.cfm?Lab=NRMRL&dirEntryId=125271

[62] Tomar M, Abdullah THA (Dec. 1994) Evaluation of chemicals to control the generation of malodorous hydrogen sulfide in waste water. Water Res 28(12):2545–2552. 10.1016/0043-1354(94)90072-8

[63] Devai I, Delaune RD (2002) Effectiveness of Selected Chemicals for Controlling Emission of Malodorous Sulfur Gases in Sewage Sludge, *Environ. Technol.*, vol. 23, no. 3, pp. 319–329, Mar. 10.1080/09593332508618412 10.1080/09593332508618412 11999994

[64] Yin R, Peng J, Sun J, Li C, Xia D, Shang C (Sep. 2021) Simultaneous removal of hydrogen sulfide, phosphate and emerging organic contaminants, and improvement of sludge dewaterability by oxidant dosing in sulfide-iron-laden sludge. Water Res 203:117557. 10.1016/J.WATRES.2021.117557 10.1016/j.watres.2021.117557 34418644

[65] Zhang L, Qiu YY, Zhou Y, Chen GH, van Loosdrecht MCM, Jiang F (Sep. 2021) Elemental sulfur as electron donor and/or acceptor: Mechanisms, applications and perspectives for biological water and wastewater treatment. Water Res 202:117373. 10.1016/J.WATRES.2021.117373 10.1016/j.watres.2021.117373 34243051

[66] Boulegue J (1978) Solubility of Elemental Sulfur in Water at 298 K, *Phosphorus and Sulfur and the Related Elements*, vol. 5, no. 1, pp. 127–128, Sep. 10.1080/03086647808069875

