# Supplementary material for: A systematic study of mitigation strategies to prevent the emission of malodours from primary settlers in wastewater treatment plants
Source: Bioprocess Biosyst Eng. 2026 May 20;49(6):1539–50. doi: 10.1007/s00449-026-03335-5 (PMC13328229; doi:10.1007/s00449-026-03335-5)

**Appendix 1**

**Fig. S1**. Concentration of the sulfur gases H_2_S (■) and CH_3_SH (●) in the headspace of the primary settler during the experiment


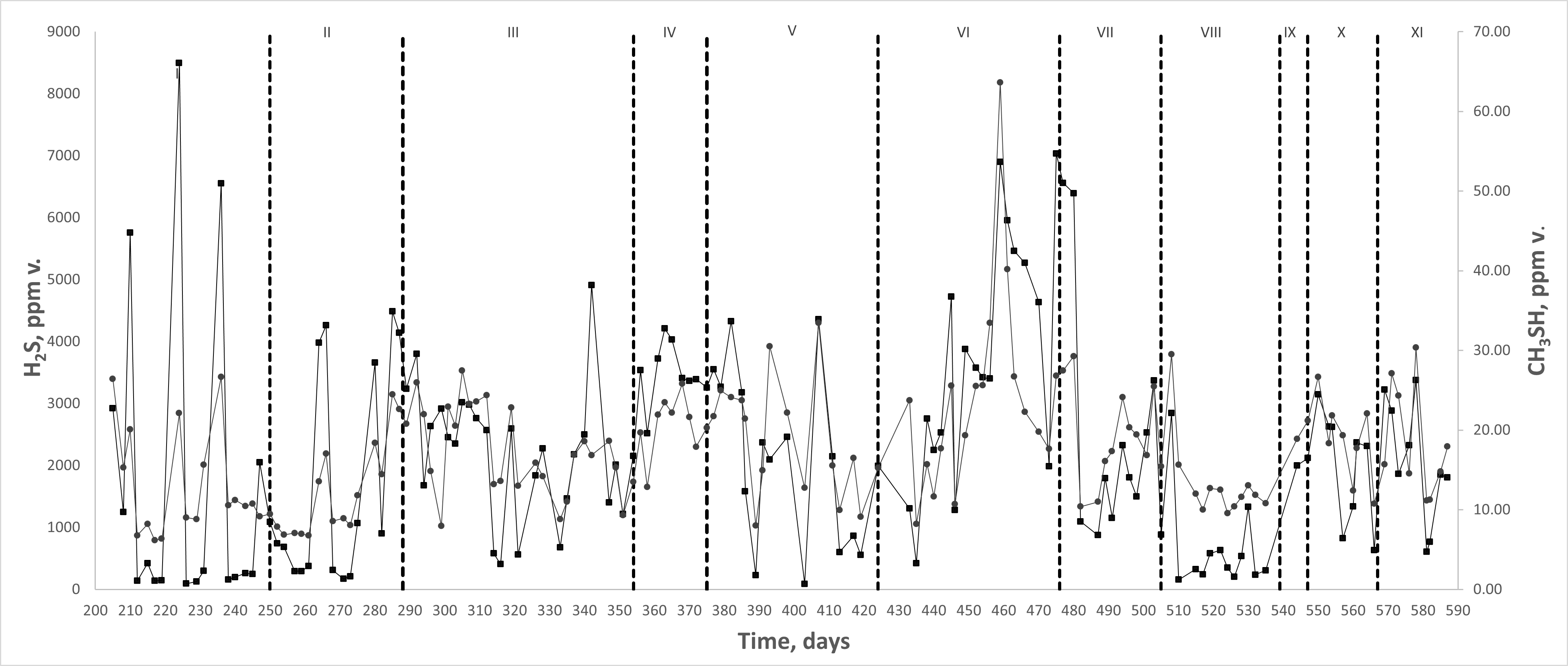


**Fig. S2.** Time course of SO_4_^2-^ concentrations (mg L^-1^) in the system influent (▲) and settler effluent (■) throughout the different treatment phases.


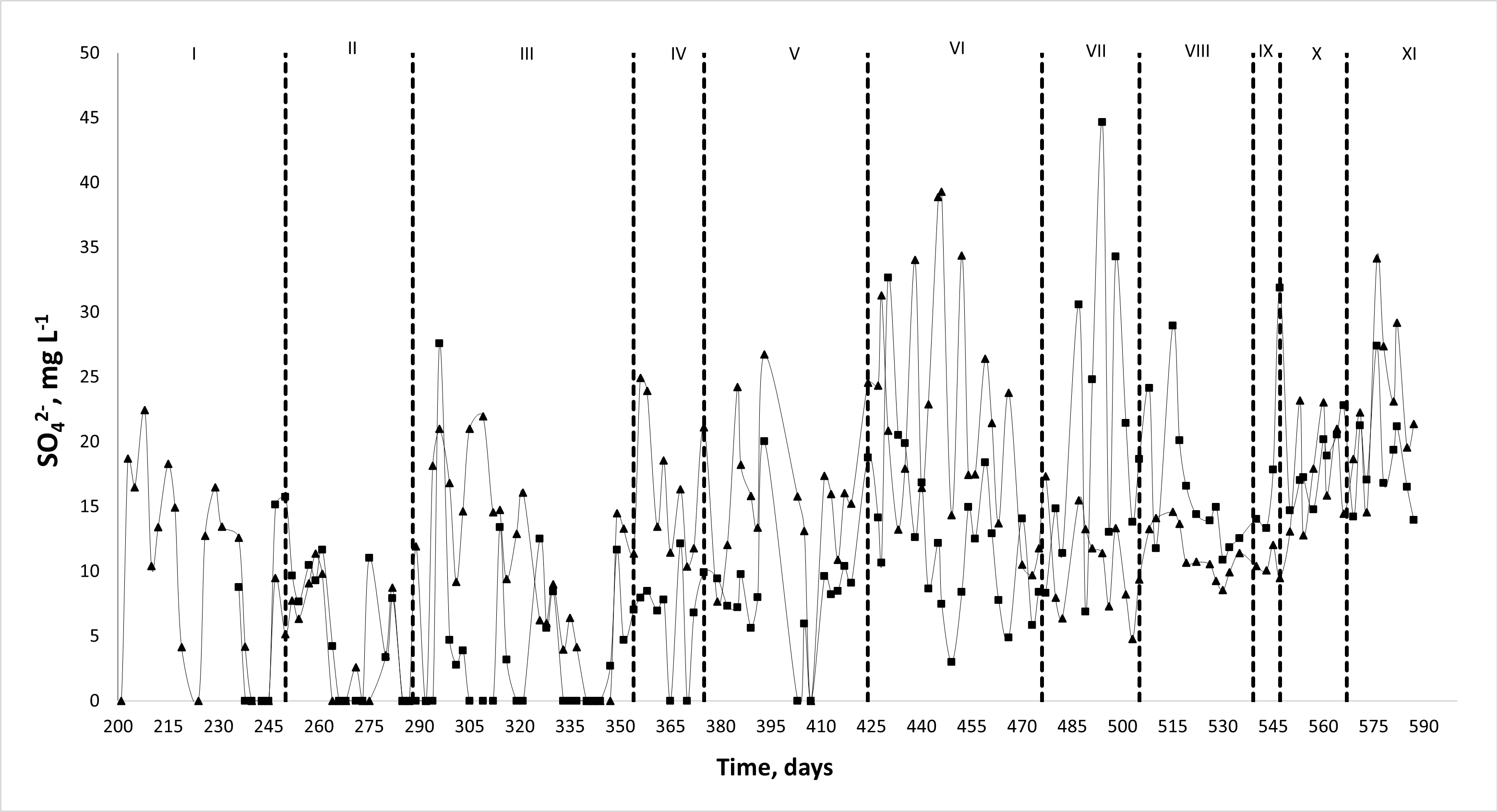


**Fig. S3.** Time course of S^2-^ concentrations in the system influent (▲) and settler effluent (■) throughout the different treatment phases.


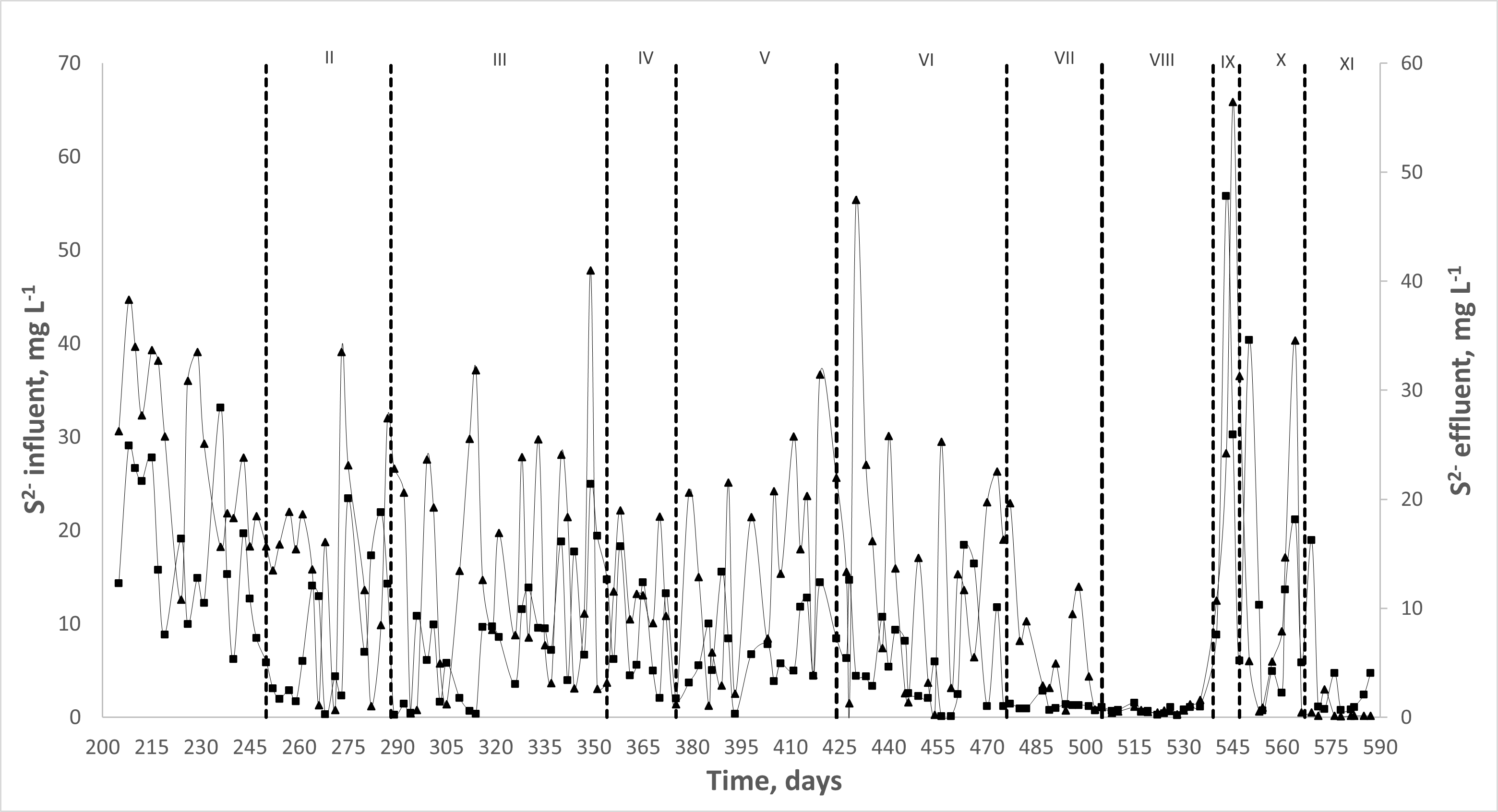


**Appendix 2**

**Table S1.** Descriptive statistics of H_2_S emissions.

| Indicator | Operational phase | | | | | | | | | |
| --- | --- | --- | --- | --- | --- | --- | --- | --- | --- | --- |
|  | **I** | **II** | **III** | **IV** | **V** | **VI** | **VII** | **VIII** | **X** | **XI** |
| Mean H_2_S concentration  (ppm) | 1685.273 | 1705.060 | 2198.663 | 3494.383 | 2105.132 | 3710.237 | 2524.450 | 644.870 | 1998.167 | 2078.351 |
| Standard Error | 608.016 | 460.298 | 204.551 | 162.278 | 347.025 | 456.575 | 573.731 | 220.121 | 289.417 | 328.467 |
| Median H_2_S concentration  (ppm) | 279.414 | 741.460 | 2311.405 | 3409.250 | 2121.490 | 3498.500 | 1801.770 | 336.605 | 2312.020 | 1862.910 |
| Standard Deviation | 2579.594 | 1782.725 | 1043.012 | 486.835 | 1388.102 | 1937.085 | 1987.461 | 762.520 | 868.251 | 985.400 |
| Sample Variance | 6654305.817 | 3178107.503 | 1087873.691 | 237008.062 | 1926827.004 | 3752299.306 | 3950002.515 | 581436.434 | 753859.473 | 971013.002 |
| Kurtosis | 2.145 | -1.519 | 0.673 | 1.413 | -1.022 | -0.817 | 1.159 | 7.048 | -0.962 | -0.974 |
| Skewness | 1.788 | 0.749 | 0.270 | -0.570 | 0.127 | 0.154 | 1.502 | 2.587 | -0.587 | -0.211 |
| Range | 8400.660 | 4318.320 | 4505.090 | 1689.820 | 4271.050 | 6612.420 | 5685.110 | 2688.030 | 2514.270 | 2772.810 |
| Minimum | 92.160 | 167.890 | 407.280 | 2519.490 | 90.300 | 423.460 | 875.470 | 155.490 | 629.590 | 605.530 |
| Maximum | 8492.820 | 4486.210 | 4912.370 | 4209.310 | 4361.350 | 7035.880 | 6560.580 | 2843.520 | 3143.860 | 3378.340 |
| Count | 18 | 15 | 26 | 9 | 16 | 18 | 12 | 12 | 9 | 9 |
| Confidence Level (95.0%) | 1282.802 | 987.240 | 421.282 | 374.214 | 739.667 | 963.290 | 1262.773 | 484.482 | 667.397 | 757.445 |
| Coefficient of variation, % | 153.07 | 104.55 | 47.44 | 13.93 | 65.94 | 52.21 | 78.73 | 118.24 | 43.45 | 47.41 |

**Table S2.** Descriptive statistics of CH_3_SH concentrations.

| Indicator | Operational phase | | | | | | | | | | | | | | | | | | | |  |
| --- | --- | --- | --- | --- | --- | --- | --- | --- | --- | --- | --- | --- | --- | --- | --- | --- | --- | --- | --- | --- | --- |
|  | **I** | | **II** | | **III** | | **IV** | | **V** | | **VI** | | **VII** | | **VIII** | | **X** | | **XI** | |  |
| Mean CH_3_SH concentration  (ppm) | | 12.957 | | 12.225 | | 17.513 | | 20.640 | | 19.010 | | 24.131 | | 19.435 | | 13.305 | | 18.930 | | 18.576 | |
| Standard Error | | 1.561 | | 1.543 | | 1.082 | | 1.228 | | 1.868 | | 2.979 | | 1.766 | | 1.545 | | 1.643 | | 2.331 | |
| Median CH_3_SH concentration  (ppm) | | 10.515 | | 8.920 | | 16.855 | | 21.620 | | 18.955 | | 23.020 | | 18.375 | | 11.920 | | 19.340 | | 15.700 | |
| Standard Deviation | | 6.621 | | 5.978 | | 5.515 | | 3.685 | | 7.472 | | 12.638 | | 6.116 | | 5.351 | | 4.929 | | 6.994 | |
| Sample Variance | | 43.836 | | 35.732 | | 30.412 | | 13.577 | | 55.835 | | 159.717 | | 37.407 | | 28.638 | | 24.296 | | 48.922 | |
| Kurtosis | | 0.081 | | -0.280 | | -0.991 | | 1.952 | | -0.611 | | 5.058 | | -0.933 | | 9.274 | | -0.017 | | -0.998 | |
| Skewness | | 1.129 | | 0.961 | | 0.016 | | -1.037 | | 0.290 | | 1.866 | | 0.153 | | 2.934 | | -0.393 | | 0.702 | |
| Range | | 20.510 | | 17.740 | | 19.520 | | 12.940 | | 25.440 | | 55.410 | | 18.883 | | 19.930 | | 15.900 | | 19.200 | |
| Minimum | | 6.150 | | 6.750 | | 7.950 | | 12.870 | | 8.020 | | 8.220 | | 10.387 | | 9.570 | | 10.770 | | 11.140 | |
| Maximum | | 26.660 | | 24.490 | | 27.470 | | 25.810 | | 33.460 | | 63.630 | | 29.270 | | 29.500 | | 26.670 | | 30.340 | |
| Count | | 18 | | 15 | | 26 | | 9 | | 16 | | 18 | | 12 | | 12 | | 9 | | 9 | |
| Confidence Level (95.0%) | | 3.292 | | 3.310 | | 2.227 | | 2.832 | | 3.982 | | 6.285 | | 3.886 | | 3.400 | | 3.789 | | 5.376 | |
| Coefficient of variation, % | | 51.10 | | 48.90 | | 31.49 | | 17.85 | | 39.31 | | 52.37 | | 31.47 | | 40.22 | | 26.04 | | 37.65 | |

**Table S3.** Descriptive statistics of S-SO_4_^2-^ concentrations in the influent of the primary settler during different treatment phases.

| Indicator | Operational phase | | | | | | | | | |
| --- | --- | --- | --- | --- | --- | --- | --- | --- | --- | --- |
|  | I | II | III | IV | V | VI | VII | VIII | X | XI |
| Mean S-SO_4_^2-^ concentration  (mg L^-1^) | 3.23 | 1.32 | 3.58 | 5.63 | 5.32 | 7.11 | 3.52 | 3.85 | 5.59 | 7.80 |
| Standard Error | 0.56 | 0.38 | 0.46 | 0.62 | 0.61 | 0.76 | 0.37 | 0.21 | 0.54 | 0.67 |
| Median S-SO_4_^2-^ concentration  (mg L^-1^) | 3.84 | 0.87 | 3.98 | 5.45 | 5.30 | 5.91 | 3.47 | 3.58 | 5.29 | 7.43 |
| Standard Deviation | 2.37 | 1.46 | 2.31 | 1.85 | 2.29 | 3.22 | 1.28 | 0.69 | 1.62 | 2.00 |
| Sample Variance | 5.64 | 2.15 | 5.32 | 3.41 | 5.24 | 10.34 | 1.65 | 0.47 | 2.63 | 3.99 |
| Kurtosis | -1.15 | -1.59 | -1.01 | -1.58 | 1.32 | -0.63 | -0.84 | -1.36 | -1.27 | -0.02 |
| Skewness | -0.07 | 0.48 | -0.16 | 0.33 | -0.61 | 0.77 | 0.28 | 0.24 | 0.11 | 0.50 |
| Range | 7.50 | 3.79 | 7.33 | 4.86 | 8.92 | 9.88 | 4.19 | 2.02 | 4.58 | 6.55 |
| Minimum | 0.00 | 0.00 | 0.00 | 3.46 | 0.00 | 3.23 | 1.59 | 2.85 | 3.16 | 4.85 |
| Maximum | 7.50 | 3.79 | 7.33 | 8.32 | 8.92 | 13.11 | 5.79 | 4.87 | 7.74 | 11.40 |
| Count | 18 | 15 | 25 | 9 | 14 | 18 | 12 | 11 | 9 | 9 |
| Confidence Level (95.0%) | 1.18 | 0.81 | 0.95 | 1.42 | 1.32 | 1.60 | 0.82 | 0.46 | 1.25 | 1.54 |

**Table S4.** Descriptive statistics of S-SO_4_^2-^ concentrations in the effluent of the primary settler during different treatment phases.

| Indicator | Operational phase | | | | | | | | | |
| --- | --- | --- | --- | --- | --- | --- | --- | --- | --- | --- |
|  | I | II | III | IV | V | VI | VII | VIII | X | XI |
| Mean S-SO^2-^_4_ concentration  (mg L^-1^) | 0.74 | 1.68 | 1.33 | 2.23 | 2.95 | 3.88 | 6.75 | 5.47 | 6.61 | 6.22 |
| Standard Error | 0.41 | 0.41 | 0.43 | 0.46 | 0.50 | 0.41 | 1.10 | 0.58 | 0.59 | 0.47 |
| Median S-SO_4_^2-^ concentration  (mg L^-1^) | 0.00 | 1.41 | 0.00 | 2.61 | 2.90 | 4.12 | 5.60 | 4.81 | 6.32 | 5.70 |
| Standard Deviation | 1.75 | 1.60 | 2.16 | 1.38 | 1.86 | 1.73 | 3.83 | 1.92 | 1.76 | 1.41 |
| Sample Variance | 3.06 | 2.55 | 4.65 | 1.89 | 3.44 | 3.00 | 14.64 | 3.68 | 3.09 | 1.98 |
| Kurtosis | 3.49 | -1.90 | 6.74 | 0.07 | 1.02 | -0.89 | 0.25 | 1.02 | 3.39 | 1.25 |
| Skewness | 2.19 | 0.10 | 2.40 | -0.89 | 0.51 | 0.19 | 0.94 | 1.33 | 1.65 | 1.07 |
| Range | 5.25 | 3.90 | 9.21 | 4.05 | 6.69 | 5.85 | 12.60 | 6.02 | 5.74 | 4.49 |
| Minimum | 0.00 | 0.00 | 0.00 | 0.00 | 0.00 | 1.00 | 2.31 | 3.64 | 4.91 | 4.66 |
| Maximum | 5.25 | 3.90 | 9.21 | 4.05 | 6.69 | 6.85 | 14.90 | 9.66 | 10.64 | 9.15 |
| Count | 18 | 15 | 25 | 9 | 14 | 18 | 12 | 11 | 9 | 9 |
| Confidence Level (95.0%) | 0.87 | 0.88 | 0.89 | 1.06 | 1.07 | 0.86 | 2.43 | 1.29 | 1.35 | 1.08 |

**Table S5.** Descriptive statistics of S^2-^ behavior in the influent of the primary settler during different treatment phases.

| Indicator | Operational phase | | | | | | | | | |
| --- | --- | --- | --- | --- | --- | --- | --- | --- | --- | --- |
|  | I | II | III | IV | V | VI | VII | VIII | X | XI |
| Mean S^2-^ concentration  (mg L^-1^) | 28.83 | 17.02 | 17.14 | 12.91 | 15.49 | 14.48 | 7.14 | 0.82 | 13.04 | 0.51 |
| Standard Error | 2.21 | 2.84 | 2.56 | 2.08 | 3.05 | 2.40 | 1.91 | 0.13 | 5.11 | 0.31 |
| Median S^2-^ concentration  (mg L^-1^) | 29.66 | 17.96 | 15.66 | 13.04 | 15.16 | 15.62 | 5.06 | 0.67 | 6.03 | 0.17 |
| Standard Deviation | 9.38 | 10.99 | 12.82 | 6.23 | 11.43 | 10.17 | 6.60 | 0.45 | 15.33 | 0.93 |
| Sample Variance | 87.91 | 120.82 | 164.35 | 38.77 | 130.57 | 103.37 | 43.59 | 0.20 | 235.01 | 0.86 |
| Kurtosis | -1.17 | -0.04 | -0.44 | 0.85 | -1.08 | -1.37 | 1.73 | 1.45 | -0.06 | 8.52 |
| Skewness | -0.02 | 0.18 | 0.47 | -0.10 | 0.36 | 0.08 | 1.31 | 1.40 | 1.20 | 2.90 |
| Range | 32.05 | 38.31 | 47.42 | 20.76 | 35.46 | 29.83 | 22.19 | 1.50 | 39.78 | 2.88 |
| Minimum | 12.61 | 0.80 | 0.40 | 1.39 | 1.24 | 0.25 | 0.72 | 0.33 | 0.52 | 0.09 |
| Maximum | 44.66 | 39.11 | 47.83 | 22.16 | 36.70 | 30.08 | 22.91 | 1.83 | 40.30 | 2.97 |
| Count | 18 | 15 | 25 | 9 | 14 | 18 | 12 | 11 | 9 | 9 |
| Confidence Level (95.0%) | 4.66 | 6.09 | 5.29 | 4.79 | 6.60 | 5.06 | 4.19 | 0.30 | 11.78 | 0.71 |

**Table S6.** Descriptive statistics of S^2-^ concentrations in the effluent of the primary settler during different treatment phases.

| Indicator | Operational phase | | | | | | | | | |
| --- | --- | --- | --- | --- | --- | --- | --- | --- | --- | --- |
|  | I | II | III | IV | V | VI | VII | VIII | X | XI |
| Mean S^2-^ concentration  (mg L^-1^) | 14.54 | 7.63 | 6.77 | 6.79 | 6.51 | 5.04 | 1.07 | 0.69 | 10.23 | 3.38 |
| Standard Error | 1.69 | 1.72 | 1.11 | 1.68 | 0.97 | 1.11 | 0.13 | 0.10 | 3.54 | 1.67 |
| Median S^2-^ concentration  (mg L^-1^) | 12.96 | 5.15 | 6.18 | 4.81 | 5.83 | 3.30 | 0.98 | 0.66 | 5.20 | 0.98 |
| Standard Deviation | 7.15 | 6.65 | 5.55 | 5.04 | 3.63 | 4.72 | 0.47 | 0.34 | 10.63 | 5.02 |
| Sample Variance | 51.12 | 44.21 | 30.86 | 25.42 | 13.19 | 22.26 | 0.22 | 0.11 | 112.90 | 25.19 |
| Kurtosis | -0.86 | -0.88 | 0.71 | -0.79 | -0.16 | 0.31 | 7.07 | 0.03 | 3.24 | 7.10 |
| Skewness | 0.51 | 0.72 | 0.94 | 0.83 | 0.47 | 1.08 | 2.42 | 0.22 | 1.75 | 2.60 |
| Range | 23.38 | 19.79 | 21.17 | 13.96 | 13.01 | 15.69 | 1.78 | 1.16 | 33.99 | 15.61 |
| Minimum | 5.04 | 0.29 | 0.21 | 1.73 | 0.32 | 0.10 | 0.63 | 0.17 | 0.60 | 0.64 |
| Maximum | 28.42 | 20.08 | 21.38 | 15.69 | 13.33 | 15.79 | 2.41 | 1.32 | 34.60 | 16.25 |
| Count | 18 | 15 | 25 | 9 | 14 | 18 | 12 | 11 | 9 | 9 |
| Confidence Level (95.0%) | 3.56 | 3.68 | 2.29 | 3.88 | 2.10 | 2.35 | 0.30 | 0.23 | 8.17 | 3.86 |

**Appendix 3**

**Fig. F1.** Photos of an open settler taken after the experiment was finished (15.07.24). Big flocks of solids could be seen floating on a liquid surface.


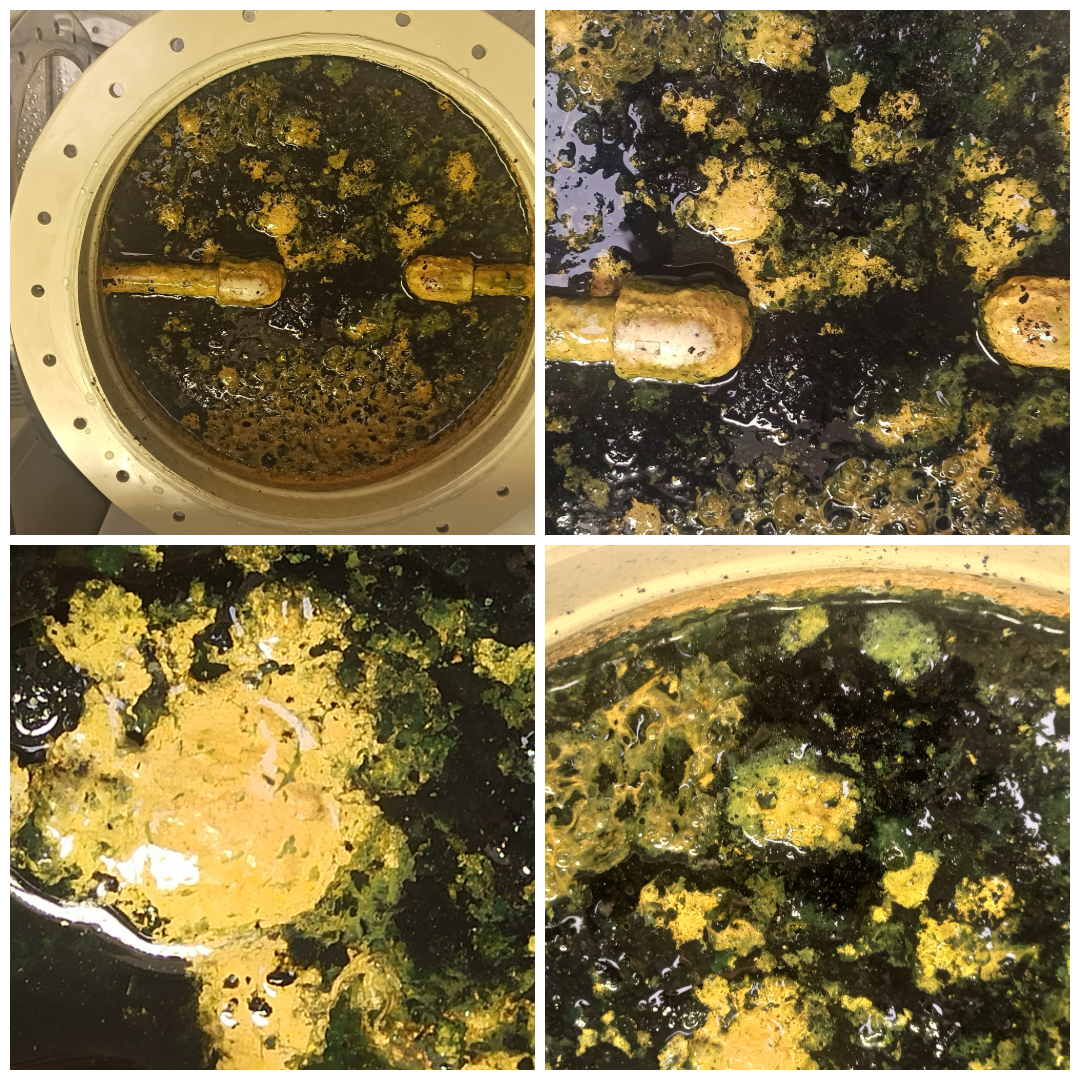

Supplement: Supplementary file 1 — Supplementary Material 1 [file 449_2026_3335_MOESM1_ESM.docx]
